# Analysis of hepatitis B virus infection in 1424 patients with different pathological types of lymphoma (2018–2022): A real‐world, retrospective study

**DOI:** 10.1002/cam4.7284

**Published:** 2024-05-16

**Authors:** Zhaoxia Li, Wei Guo, Yangzhi Zhao, Haotian Wang, Jing Guo, Zhe Li, Bowen Wang, Luming Cao, Jihong Xu, Ken H. Young, Ou Bai

**Affiliations:** ^1^ Department of Hematology The First Hospital of Jilin University Changchun Jilin China; ^2^ Department of Hematopathology Duke Cancer Institute, Duke University Medical Center Durham North Carolina USA

**Keywords:** aggressive B‐cell, non‐Hodgkin's lymphoma, hepatitis B virus, indolent B‐cell non‐Hodgkin's lymphoma, non‐Hodgkin's lymphoma

## Abstract

**Objective:**

Recent studies have found a high prevalence of hepatitis B virus (HBV) infection in patients with non‐Hodgkin's lymphoma (NHL), especially B‐cell non‐Hodgkin's lymphoma (B‐NHL). However, most studies did not classify it and analyze the correlation between HBV and its various subtypes.

**Methods:**

The authors retrospectively analyzed 1424 patients with lymphoma. Differences in the prevalence of HBV infection in patients with different pathological types of lymphoma were analyzed. The clinical characteristics, progression‐free survival (PFS), and overall survival (OS) of HBV‐positive and negative B‐NHL subtypes were compared according to HBV infection.

**Results:**

The HBV infection rate in NHL patients was 7.65%, which was higher than that in HL patients (2.59%, *p* < 0.05). The HBV infection rate in the B‐NHL was higher than that in the T‐cell non‐Hodgkin's lymphoma (T‐NHL) (8.14% vs. 4.95%). The HBV infection rate in the aggressive B‐NHL was similar to that of the indolent B‐NHL (8.30% vs. 7.88%), and the highest HBV infection rates were found in diffuse large B‐cell lymphoma and chronic lymphocytic leukemia/small lymphocytic lymphoma, but no significant differences in clinical characteristics, PFS, and OS were seen between HBV‐positive and negative patients in the two subtypes.

**Conclusions:**

There was an association between HBV infection and the development of NHL and HBV infection may play a role in the pathogenesis of B‐NHL, but not T‐NHL.

## 1 INTRODUCTION

Lymphoma is the most common malignant tumor in the blood system, originating in lymphohematopoietic tissues. GLOBOCAN 2020 data showed that the incidence of lymphoma accounted for 50% of hematological tumors and 3%–5% of all tumors.^1^ Lymphoma classification is complex and highly heterogeneous. Based on histopathology, they can be divided into Hodgkin's lymphoma (HL) and non‐Hodgkin's lymphoma (NHL), of which NHL is dominant, accounting for about 80%–85% of all lymphomas. Based on the cellular immunophenotype, NHL can be divided into B‐cell non‐Hodgkin's lymphoma (B‐NHL) and T‐cell non‐Hodgkin's lymphoma (T‐NHL), of which B‐NHL is dominant, accounting for about 85%–90% of NHL. Based on the malignant degree of the disease, B‐NHL can be further divided into aggressive B‐NHL and indolent B‐NHL.

Hepatitis B virus (HBV) is a double‐stranded DNA virus with hepatotropic and lymphotropic characteristics that has attracted increasing attention in recent years as a possible cause of lymphoma. Some studies have found that HBV may be correlated with the occurrence of NHL. For example, NHL patients had higher rates of HBV infection than healthy people and those with solid tumors other than primary liver cancer.^2^, ^3^, ^4^ The rate of HBV infection in B‐NHL was higher than in the T‐NHL.^5^ However, there are many types of NHL, and the correlation between HBV infection and various subtypes of B‐NHL and even T‐NHL is still unclear, and the conclusions of domestic and international studies also lack consistency. Moreover, current studies mainly focused on the correlation between HBV and B‐NHL, especially aggressive B‐NHL, while very few studies have classified all types of lymphoma and explored the correlation between HBV and each subtype. Therefore, based on the clinical data of lymphoma patients in our academic center from January 2018 to December 2022 (including HL, B‐NHL, T‐NHL, and their subtypes), this study retrospectively analyzed the rate of HBV infection in patients with different pathological types of lymphoma, explored the correlation between HBV infection and different subtypes of lymphoma, in the hope of providing a basis for the future related clinical and basic research.

## 2 MATERIALS AND METHODS

### 2.1 Patient selection and data collection

We collected a total of 1466 cases of newly diagnosed lymphoma between 1 January 2018 and 31 December 2022. 42 patients were excluded from the study due to unclassified pathological types and incomplete clinical data. Finally, 1424 patients were included.

The clinical and histological data were collected from these patients, including age, gender, pathology type, Ann‐Arbor stage, HBV seromarkers, etc. The follow‐up information was obtained through a medical record review or telephone survey. HBV infection referred to a positive detection of hepatitis B surface antigen (HBsAg) at the time of lymphoma diagnosis. Progression‐free survival (PFS) was the period from the date of diagnosis to tumor progression. Overall survival (OS) referred to the period from the date of diagnosis to death due to any cause. The data cutoff date for this study was October 2023.

### 2.2 Statistical analysis

Descriptive statistics were conducted for the patient's baseline characteristics, which are presented as frequency counts and percentages or medians and ranges. Categorical parameters are presented as the number and percentage of patients. The chi‐squared test was used for comparisons of enumeration data. Survival analysis was performed using the Kaplan–Meier method and the log‐rank test. All the statistical analyses were performed with SPSS statistics 25.0 and Graphpad prism 6.0. A two‐tailed *p* value<0.05 was considered statistically significant.

## 3 RESULTS

### 3.1 Patient characteristics

**FIGURE 1:**
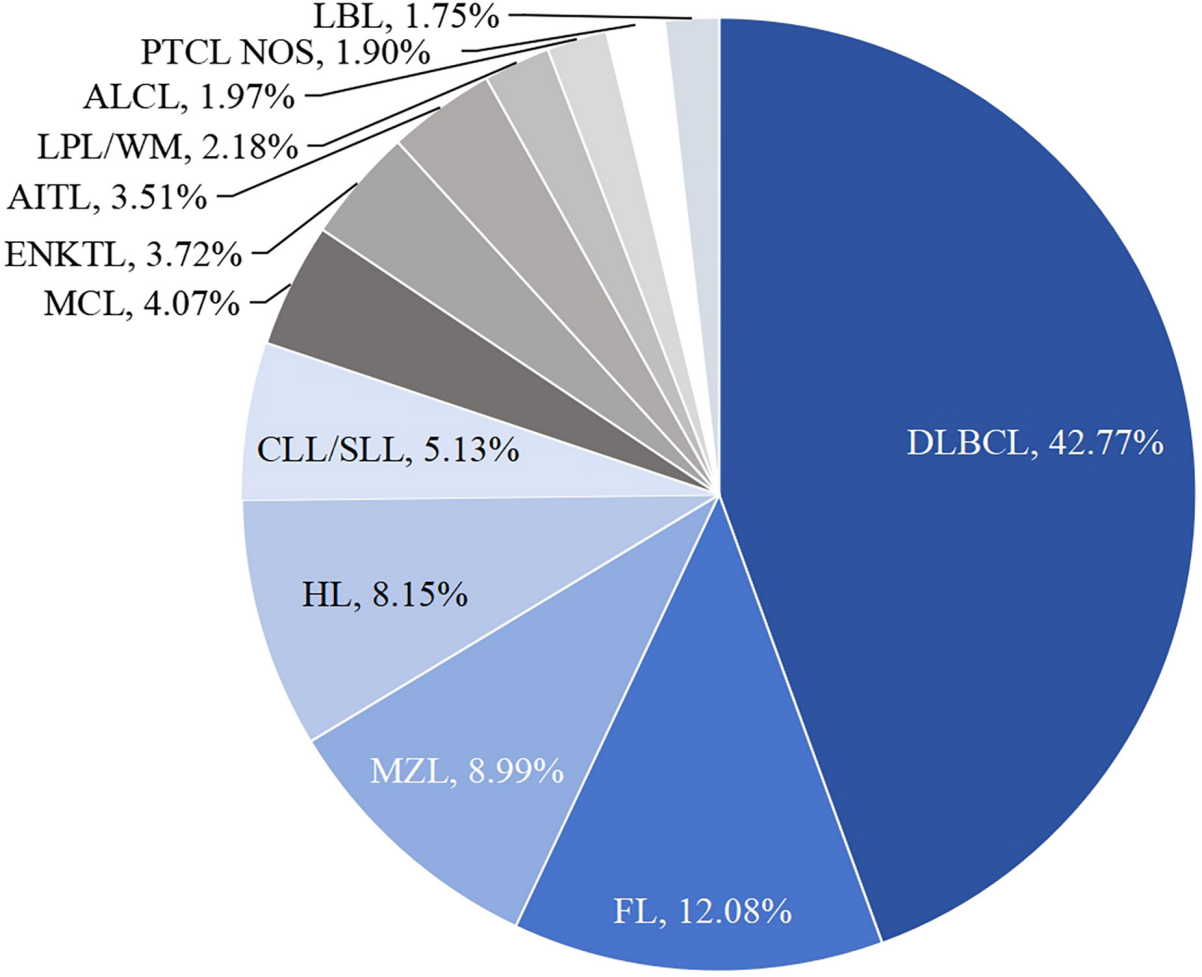
Distribution of the major pathological types in 1424 patients with lymphoma. HL, Hodgkin's lymphoma; LBL, lymphoblastic lymphoma; CLL/SLL, chronic lymphocytic leukemia/small lymphocytic lymphoma; DLBCL, diffuse large B‐cell lymphoma; FL, follicular lymphoma; LPL/WM, lymphoplasmacytic lymphoma/Waldenström macroglobulinemia; MCL, mantle cell lymphoma; MZL, marginal zone lymphoma; AITL, angioimmunoblastic T‐cell lymphoma; ALCL, anaplastic large cell lymphoma; ENKTL, extranodal NK/T‐cell lymphoma; PTCL NOS, peripheral T‐cell lymphoma, not otherwise specified.

The baseline characteristics of the 1424 lymphoma patients are summarized in Table 1. The number of HL and NHL cases was 116 and 1308, respectively, with a composition ratio of 8.15% and 91.85% of all lymphomas. Among the 1308 NHL patients, lymphoblastic lymphoma (LBL), mature B‐cell lymphoma (BCL), and mature T/NK‐cell lymphoma (T/NKCL) were 25, 1102, and 181 cases, respectively, with a composition ratio of 1.75%, 77.39%, and 12.71% of all lymphomas. Diffuse large B‐cell lymphoma (DLBCL) was the most common subtype of all lymphomas and BCL, accounting for a proportion of up to 42.77% of all lymphoma patients. Extranodal NK/T‐cell lymphoma (ENKTL) and angioimmunoblastic T‐cell lymphoma (AITL) were the most common subtypes of T/NKCL. The distribution of the major pathological types in 1424 patients with lymphoma is shown in Figure 1. Overall, the median age of patients was 59 (12–94) years, and the ratio of ≤60 years of age to ≥60 years was 1.2:1, with a slight predominance of individuals ≤60 years. The ratio of male to female was 1.3:1, with male predominating. The majority of patients (66.78%) were already in the advanced stage (Stages III‐IV) at the time of diagnosis, with a ratio of 2:1 compared to those at the early stage (Stages I‐II).

**TABLE 1:** Baseline characteristics of 1424 patients with lymphoma.

Characteristics	Number (%)
Histology	
HL	116 (8.15)
cHL	115 (8.08)
NLPHL	1 (0.07)
NHL	1308 (91.85)
LBL	25 (1.75)
Mature BCL	1102 (77.39)
BL	12 (0.84)
B‐LPD	10 (0.70)
CLL/SLL	73 (5.13)
DLBCL	609 (42.77)
FL	172 (12.08)
HCL	5 (0.35)
LPL/WM	31 (2.18)
MCL	58 (4.07)
MZL	128 (8.99)
Others	4 (0.28)
Mature T/NKCL	181 (12.71)
AITL	50 (3.51)
ALK+ALCL	18 (1.27)
ALK‐ALCL	10 (0.70)
EATL	6 (0.42)
ENKTL	53 (3.72)
MF/SS	4 (0.28)
PTCL NOS	27 (1.90)
T‐LGL	4 (0.28)
T‐LPD	4 (0.28)
Others	5 (0.35)
Age	
Median age	59 (12–94 years)
>60 years	657 (46.14)
≤60 years	767 (53.86)
Sex	
Male	806 (56.60)
Female	618 (43.40)
Stage	
I‐II	473 (33.22)
III‐IV	951 (66.78)

Abbreviations: AITL, angioimmunoblastic T‐cell lymphoma; ALK+ALCL, anaplastic lymphoma kinase positive anaplastic large cell lymphoma; ALK−ALCL, anaplastic lymphoma kinase negative anaplastic large cell lymphoma; BCL, mature B‐cell lymphoma; BL, Burkitt lymphoma; B‐LPD, B lymphocyte proliferative disease; cHL, classic Hodgkin lymphoma; CLL/SLL, chronic lymphocytic leukemia/small lymphocytic lymphoma; DLBCL, diffuse large B‐cell lymphoma; EATL, enteropathy‐associated T‐cell lymphoma; ENKTL, extranodal NK/T‐cell lymphoma; FL, follicular lymphoma; HCL, hairy cell leukemia; HL, Hodgkin's lymphoma; LBL, lymphoblastic lymphoma; LPL/WM, lymphoplasmacytic lymphoma/Waldenström macroglobulinemia; MCL, mantle cell lymphoma; MF/SS, mycosis fungoides/Sézary syndrome; MZL, marginal zone lymphoma; NHL, non‐Hodgkin's lymphoma; NLPHL, nodular lymphocyte‐predominant Hodgkin lymphoma; PTCL NOS, peripheral T‐cell lymphoma, not otherwise specified; T‐LGL, T large granular lymphocyte; T‐LPD, T lymphocyte proliferative disease; T/NKCL, T/NK‐cell lymphoma.

### 3.2 HBV infection in different pathological types of lymphoma

Table 2 summarizes the HBV infection rates of different pathological subtypes of lymphoma. Among 1424 patients, the HBV infection rate was 7.65% in the NHL group, which was higher than that in the HL group (2.59%) (*p* < 0.05). Among NHL patients, the HBV infection rate was 8.14% in the B‐NHL group, which was higher than that in the T‐NHL group (4.95%) (*p* = 0.117). Among B‐NHL patients, the HBV infection rate was 8.30% in the aggressive B‐NHL group, which was similar to that in the indolent B‐NHL group (7.88%) (*p* = 0.804). Among T‐NHL patients, the HBV infection rate in the four major pathological subtypes was, in descending order, ALCL (7.14%), AITL (6.00%), PTCL NOS (3.70%), and ENKTL (1.89%).

**TABLE 2:** HBV infection rates in different pathological types of lymphoma.

Histology	Number (%)
HL (*n* = 116)	3 (2.59)
NHL (*n* = 1308)	100 (7.65)
B‐NHL (*n* = 1106)	90 (8.14)
Aggressive B‐NHL (*n* = 687)	57 (8.30)
Indolent B‐NHL (*n* = 419)	33 (7.88)
T‐NHL (*n* = 202)	10 (4.95)
AITL (*n* = 50)	3 (6.00)
ALCL (*n* = 28)	2 (7.14)
ENKTL (*n* = 53)	1 (1.89)
PTCL NOS (*n* = 27)	1 (3.70)
Others (*n* = 44)	3 (6.82)

Abbreviations: AITL, angioimmunoblastic T‐cell lymphoma; ALCL, anaplastic large cell lymphoma; B‐NHL, B‐cell non‐Hodgkin's lymphoma; ENKTL, extranodal NK/T‐cell lymphoma; HBV, hepatitis B virus; HL, Hodgkin's lymphoma; NHL, non‐Hodgkin's lymphoma; PTCL NOS, peripheral T‐cell lymphoma, not otherwise specified; T‐NHL, T‐cell non‐Hodgkin's lymphoma.

**FIGURE 2:**
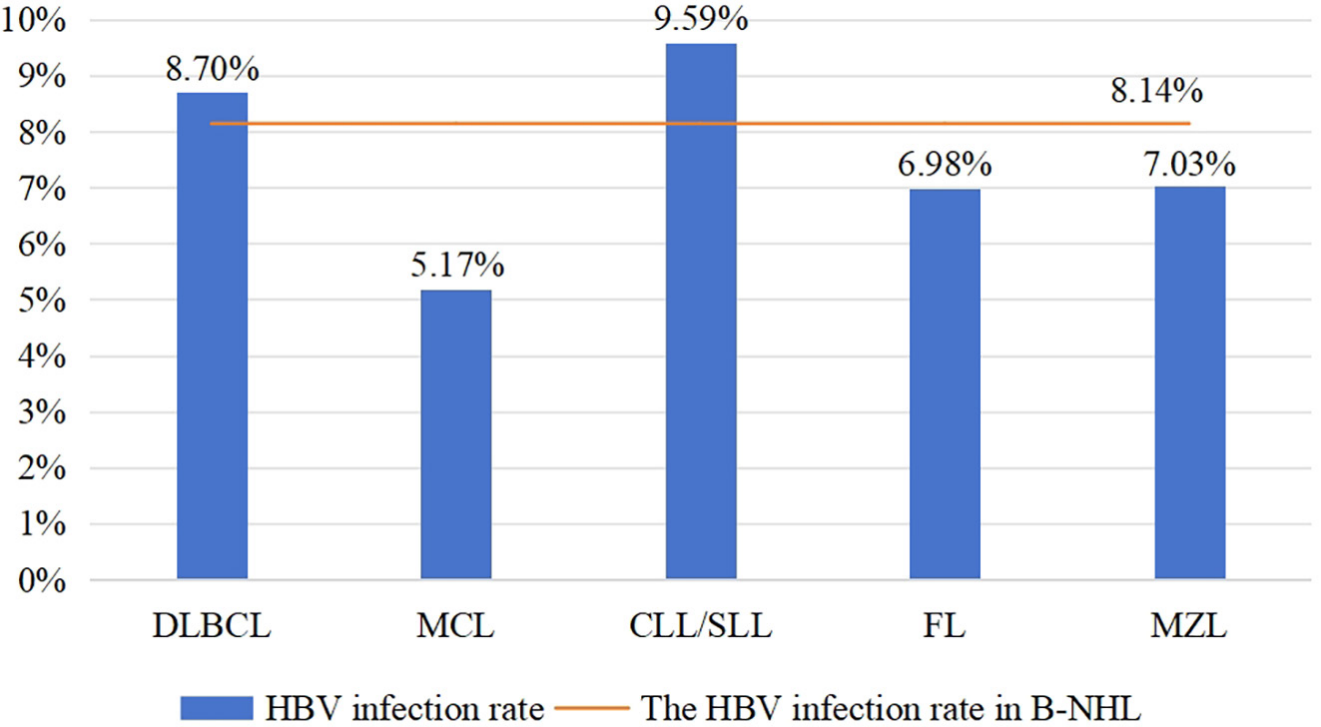
HBV infection rates of different B‐NHL subtypes. HBV, hepatitis B virus; B‐NHL, B‐cell non‐Hodgkin's lymphoma; DLBCL, diffuse large B‐cell lymphoma; MCL, mantle cell lymphoma; CLL/SLL, chronic lymphocytic leukemia/small lymphocytic lymphoma; FL, follicular lymphoma; MZL, marginal zone lymphoma.

Figure 2 further shows the HBV infection rates of the five major pathological subtypes in B‐NHL. In the aggressive B‐NHL group, DLBCL patients had the highest HBV infection rate of 8.70% (53/609), and MCL patients had an HBV infection rate of 5.17% (3/58). In the indolent B‐NHL group, CLL/SLL patients had the highest prevalence of HBV infection of 9.59% (7/73), MZL patients had a prevalence of HBV infection of 7.03% (9/128), and FL patients had a prevalence of HBV infection of 6.98% (12/172). The HBV infection rates of all subtypes of aggressive B‐NHL showed no statistical significance compared with those of all subtypes of indolent B‐NHL (all *p* > 0.05).

### 3.3 Comparison of clinical characteristics and survival analysis of HBV‐positive and HBV‐negative B‐NHL subtypes

In B‐NHL, the rate of HBV infection in DLBCL and CLL/SLL was higher than that in overall B‐NHL and overall NHL, and the following analysis focused on the clinical characteristics and survival differences in both the HBV‐positive and HBV‐negative groups of the 2 subtypes.

**FIGURE 3:**
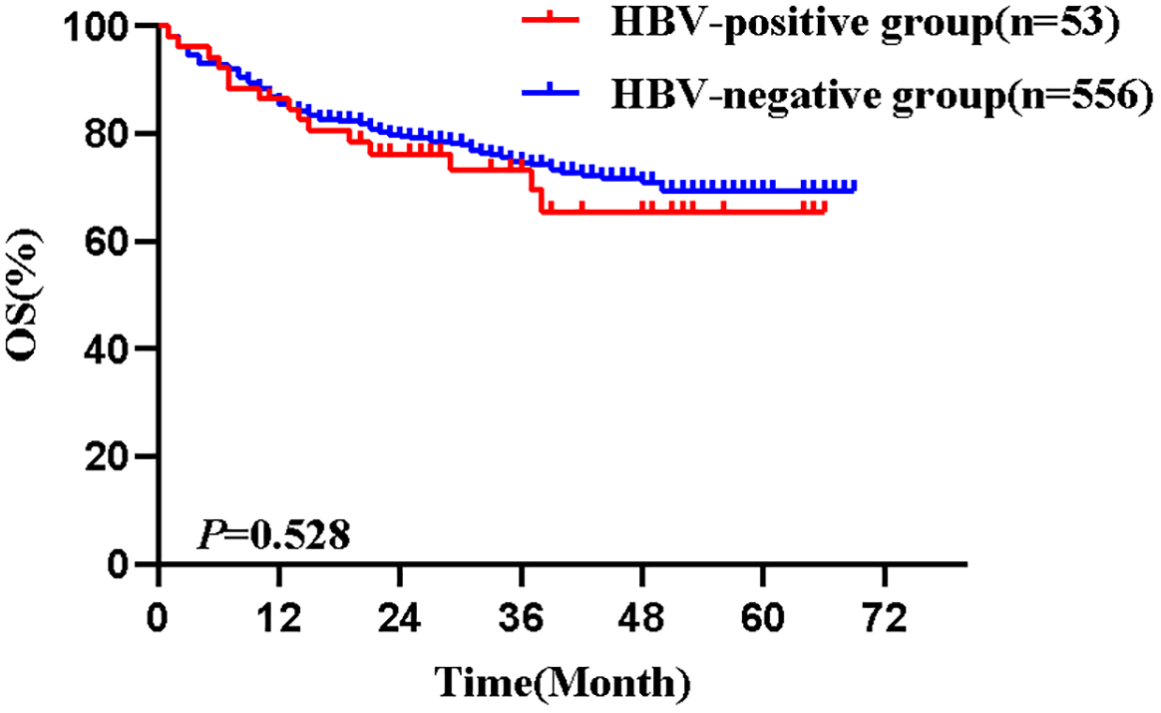
Comparison of OS in HBV‐positive and HBV‐negative DLBCL patients. DLBCL, diffuse large B‐cell lymphoma; HBV, hepatitis B virus; OS, overall survival.

Among DLBCL patients, 53 were HBV‐positive and 556 were HBV‐negative. Table 3 summarizes the distribution of clinical characteristics in two groups of patients. There were no statistical differences in both groups of patients in terms of age, gender, staging, and international prognostic index (IPI) score (all *p* > 0.05). The 609 DLBCL patients were followed up to October 2023, with a median follow‐up time of 35 months. Median OS (mOS) was not reached in the HBV‐positive group, with a 1‐year OS of 84.9% and a 2‐year OS of 74.9%. mOS was not reached in the HBV‐negative group, with a 1‐year OS of 83.6% and a 2‐year OS of 77.6%. There was no statistical significance in OS in the two groups (*p* = 0.528) (Figure 3).

**TABLE 3:** Comparison of clinical characteristics between HBV‐positive and HBV‐negative DLBCL patients (Number [%]).

Group	Number	Age		Sex	
		≤60 years	>60 years	Male	Female
HBV‐positive group	53	28 (52.83)	25 (47.17)	28 (52.83)	25 (47.17)
HBV‐negative group	556	270 (48.56)	28 (51.44)	29 (52.34)	265 (47.66)

Group	Number	Stage		IPI score	
		I‐II	III‐IV	0–2	3–5
HBV‐positive group	53	15 (28.30)	38 (71.70)	30 (56.60)	23 (43.40)
HBV‐negative group	556	192 (34.53)	364 (65.47)	287 (51.62)	269 (48.38)

Abbreviations: HBV, hepatitis B virus; DLBCL, diffuse large B‐cell lymphoma; IPI, international prognostic index.

**FIGURE 4:**
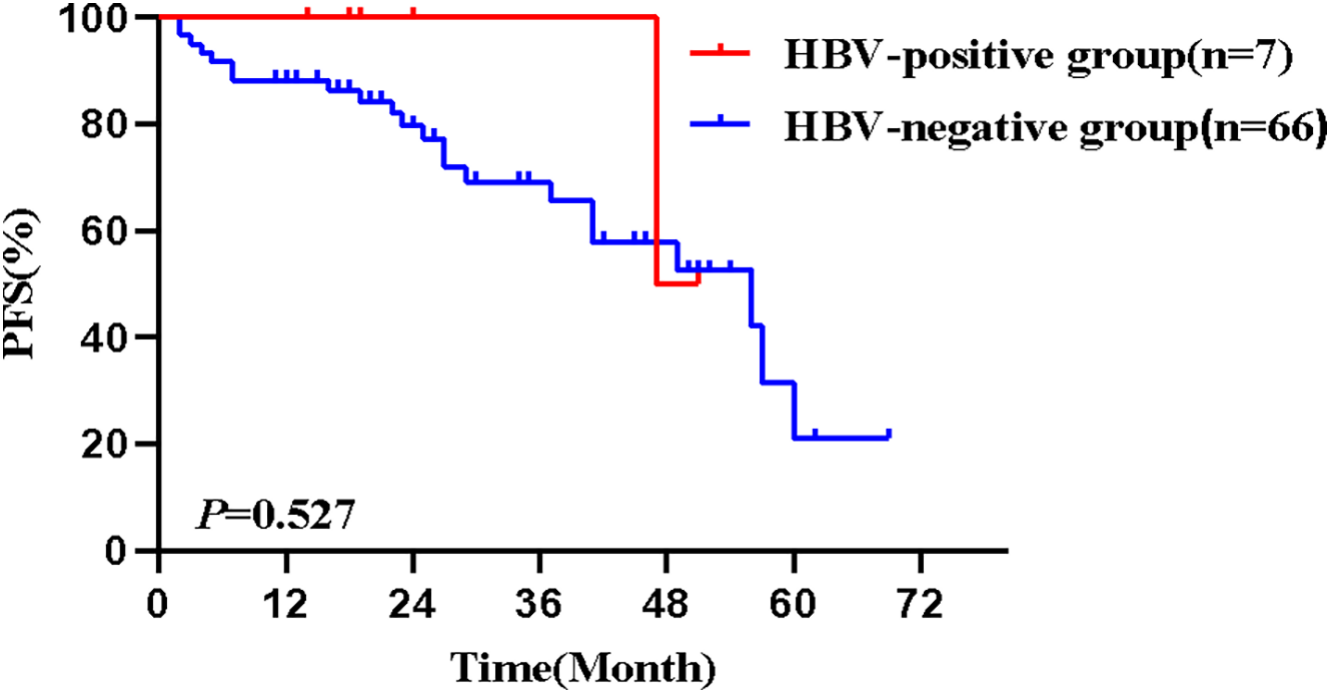
Comparison of PFS in HBV‐positive and HBV‐negative CLL/SLL patients. CLL/SLL, chronic lymphocytic leukemia/small lymphocytic lymphoma; HBV, hepatitis B virus; PFS, progression‐free survival.

Among CLL/SLL patients, 7 were HBV‐positive and 66 were HBV‐negative. Table 4 summarizes the distribution of clinical characteristics in two groups of patients. The proportion of males in the HBV‐positive group was higher than that in the HBV‐negative group, with a statistical significance (*p* = 0.04), but there were no statistical significances in terms of age and stage (all *p* > 0.05). The 73 CLL/SLL patients were followed up to October 2023, with a median follow‐up time of 34 months. The median PFS (mPFS) in the HBV‐positive group was 47 months, with a 1‐year PFS of 85.7% and a 2‐year PFS of 85.7%. mPFS in the HBV‐negative group was 49 months, with a 1‐year PFS of 80.3% and a 2‐year PFS of 72.5%. There was no statistical significance in PFS in the two groups (*p* = 0.527) (Figure 4).

**TABLE 4:** Comparison of clinical characteristics between HBV‐positive and HBV‐negative CLL/SLL patients (Number [%]).

Group	Number	Age		Sex		Stage	
		≤60 years	>60 years	Male	Female	I‐II	III‐IV
HBV‐positive group	7	2 (28.57)	5 (71.43)	7 (100)	0 (0)	7 (100)	0 (0)
HBV‐negative group	66	22 (33.33)	44 (66.67)	34 (51.52)	32 (48.48)	54 (81.82)	12 (18.18)

Abbreviations: CLL/SLL, chronic lymphocytic leukemia/small lymphocytic lymphoma; HBV, hepatitis B virus.

## 4 DISCUSSION

Lymphoma is a global disease with complex biological characteristics, and in recent years, its morbidity and mortality have been among the highest worldwide.^1^, ^6^, ^7^ In addition, the distribution of lymphoma subtypes varies among different countries and regions due to different living environments, lifestyles, and economic conditions. In this study, the proportion of HL and NHL patients was 8.15% and 91.85%, respectively, which were similar to those reported in other regions of China and Japan.^8^, ^9^, ^10^, ^11^ The distribution of the subtypes of B‐NHL and T‐NHL was also similar to the above findings. However, the ratio of mature BCL and mature T/NKCL was 6:1, which was significantly higher than that in the above studies, and this difference may be related to epidemiological factors such as regional economy, population distribution, and sample size. Moreover, the proportion of T‐NHL in the present study was significantly higher than that in Western countries.^12^, ^13^ It was in line with the epidemiological feature of higher T/NKCL proportion in Asian countries.^14^ As for gender and age, the male‐to‐female ratio in the present study was 1.3:1, with a median age of 59 years, a result that remained similar to the literature.^8^, ^9^, ^10^, ^11^ In general, the epidemiological characteristics of lymphoma in our academic center were similar to those in other parts of China and other Asian countries, but there were some differences from Western countries.

Numerous studies have confirmed the important role of pathogenic microorganisms in the lymphoma. For example, the Epstein–Barr virus (EBV) is closely related to the development of T/NKCL and BL, and the human T‐lymphotropic virus 1 (HTLV‐1) is a pathogenic factor for adult T‐cell leukemia/lymphoma (ATL). Recent studies have shown that there is also a correlation between HBV and lymphoma, especially NHL (Table 5). Kadry et al.^15^ found that the prevalence of HBV infection was significantly higher in NHL than in healthy controls and HL. Two other studies not only verified the findings of Kadry but also found that B‐NHL had a significantly higher HBsAg‐positive rate than T‐NHL, and HBV infection increased the risk of B‐NHL.^16^, ^17^ Other types of cohort studies have reached similar conclusions.^18^, ^19^ Our study also found that the prevalence of HBV infection was higher in the NHL group than in the HL group and that the B‐NHL group had a 1.6 times higher HBV infection rate than the T‐NHL group. All of these suggeste that HBV infection is associated with the development of NHL, especially B‐NHL. However, clinical studies and the study of pathogenic mechanisms at the molecular level remain inadequate and need to be further explored in the future.

**TABLE 5:** Summary of studies addressing the correlation between HBV infection and NHL.

Authors	Country/Region	Year	Study cohort	Results	Reference
Kadry DY. et al.	Egyptian	2023	NHL: 71 cases; HL: 9 cases; control (healthy volunteers): 100 cases	The positive rate of HBsAg: control: 1%; HL: 0%; NHL: 8.5%; (*p* < 0.001)	[15]
Li M. et al.	China	2020	NHL: 411 cases; control (healthy volunteers): 957 cases	Prevalence and OR of HBsAg in cases: control: 7.11%; NHL: 19.22% (OR 3.11, 95% CI: 2.20–4.41, *p* < 0.001); B‐NHL: 20.43% (OR 3.36, 95% CI: 2.33–4.84, *p* < 0.001); T‐NHL: 14% (OR 2.13, 95% CI: 0.92–4.92, *p* = 0.07)	[16]
Zhou X. et al.	China	2019	NHL: 3502 cases; control (general patients): 7004 cases	Prevalence and OR of HBsAg in cases: control: 8.8%; NHL: 14.9% (OR 1.83, 95% CI: 1.61–2.07, *p* < 0.001); B‐NHL: 17% (OR 2.14, 95% CI: 1.87–2.44, *p* < 0.001); T‐NHL: 9.4% (OR 1.08, 95% CI: 0.86–1.36, *p* = 0.51)	[17]
Lai YR. et al.	Taiwan	2022	HBV patients: 37656 cases; control (general patients): 270785 cases	The incidence rate of NHL: control: 0.1%; HBV patients: 0.22% (HR 2.49, 95% CI: 1.94–3.19, *p* < 0.001)	[19]

Abbreviations: B‐NHL, B‐cell non‐Hodgkin's lymphoma; HBsAg, hepatitis B surface antigen; HBV, hepatitis B virus; HL, Hodgkin's lymphoma; HR, Hazard Ratio; NHL, non‐Hodgkin's lymphoma; OR, Odds Ratio; T‐NHL, T‐cell non‐Hodgkin's lymphoma.

Most studies supported a correlation between HBV and B‐NHL, but there were few comparative studies on HBV infection in different subtypes of B‐NHL (Table 6). Marcucci et al.^20^ found that both aggressive and indolent B‐NHL had significantly higher HBsAg positive rates than the control group, but there was no significant difference between the two groups. However, other studies showed that the rate of HBsAg positivity in the aggressive B‐NHL was higher than in the indolent B‐NHL, so it was speculated that the aggressive group was more closely related to HBV infection in the B‐NHL.^21^, ^22^ In this study, the prevalence of HBV infection in aggressive B‐NHL was similar to indolent B‐NHL, which was similar to the results in the literature.^20^ In a further subtype analysis, Kang et al.^3^ found that DLBCL had a significantly higher HBsAg positive rate than other subtypes, and the positive rates of HBsAg in FL and NMZL also increased significantly, so it was speculated that HBV infection was associated with all three subtypes, and this result also supported the possible correlation between HBV and B‐NHL. However, both Becker and Fwu's studies have shown that HBV infection was not correlated with FL and CLL/SLL. In this study, HBV infection rates of DLBCL and CLL/SLL were higher than those of other subtypes and B‐NHL as a whole, and MCL, FL and MZL were also higher, but the differences among groups were not statistically significant. It was also worth noting that among the T‐NHL in this study, the HBV infection rates in AITL and ALCL were also higher than those in overall T‐NHL, a finding consistent with that reported by Zhou et al.^17^ It is still controversial whether HBV infection is related to B‐NHL subtypes or even T‐NHL subtypes, and we look forward to more, larger, and multicenter real‐world studies to further explore the potential link between HBV and NHL.

**TABLE 6:** Summary of studies addressing the correlation between HBV infection and B‐NHL.

Authors	Country/Region	Year	Study cohort	Results	Reference
Marcucci F. et al.	Italy	2006	B‐NHL: 399 cases (aggressive B‐NHL: 230 cases, indolent B‐NHL: 169 cases); control (general patients): 392 cases	Prevalence and AOR of HBsAg in cases: control: 2.8%; B‐NHL: 8.5% (AOR 3.67, 95% CI: 1.75–7.66); aggressive B‐NHL: 8.7% (AOR 3.75, 95% CI: 1.70–8.28) indolent B‐NHL: 8.3% (AOR 3.64, 95% CI: 1.55–8.64)	[20]
Wang C. et al.	China	2017	Aggressive B‐NHL: 373 cases; indolent B‐NHL: 255 cases	The positive rate of HBsAg: aggressive B‐NHL: 10.46%, indolent B‐NHL: 5.09%, *p* = 0.003	[21]
Xiong WJ. et al.	China	2016	Aggressive B‐NHL: 148 cases; indolent B‐NHL: 733 cases	The positive rate of HBsAg: aggressive B‐NHL: 14.2%, indolent B‐NHL: 7.9%, *p* = 0.015	[22]
Kang J. et al.	Korean	2011	Lymphoma: 2679 cases (DLBCL: 930 cases, FL 48 cases, NMZL: 44 cases); control (healthy volunteers): 915,562 cases	Prevalence and OR of HBsAg in cases: control: 4.1%; DLBCL: 16.8% (OR 4.8, 95% CI: 3.9–5.8, *p* < 0.001); FL: 20.8% (OR 5.6, 95% CI: 2.7–11.4, *p* < 0.001); NMZL: 13.6% (OR 4, 95% CI: 1.6–9.5, *p* < 0.001);	[3]
Becker N. et al.	European	2012	B‐NHL: 1136 cases (DLBCL: 350 cases, FL 157 cases, CLL: 227 cases); control (general patients): 1375 cases	Prevalence and OR of HBsAg in cases: control: 0.9%; DLBCL: 1.4% (OR 1.5, 95% CI: 0.47–4.82, *p* = 0.495); FL: 1.3% (OR 1.85, 95% CI: 0.39–8.82, *p* = 0.441); CLL: 0.9% (OR 0.83, 95% CI: 0.1–6.77, *p* = 0.86);	[23]
Fwu CW. et al.	Taiwan	2011	① HBsAg‐negative: 1492409 cases; ② HBsAg‐positive: 289992 cases	The incidence rate* of NHL subtype: DLBCL: ① 0.60% (0.47–0.77); ② 1.81% (1.31–2.48) FL: ① 0.15% (0.09–0.24); ② 0.14% (0.05–0.44) CLL: ① 0.07% (0.03–0.14); ② 0.14% (0.05–0.44)	[24]

Abbreviations: AOR, Adjusted Odds Ratio; B‐NHL, B‐cell non‐Hodgkin's lymphoma; CLL, chronic lymphocytic leukemia; DLBCL, diffuse large B‐cell lymphoma; FL, follicular lymphoma; HBsAg, hepatitis B surface antigen; NHL, non‐Hodgkin's lymphoma; NMZL, nodal marginal zone lymphomaMCL, mantle cell lymphoma.

* Per 100,000 person‐years.

Several studies in recent years have shown that patients with HBsAg‐positive DLBCL were younger and had a higher proportion of stage III/IV and a poorer prognosis compared with the HBsAg‐negative group.^25^, ^26^ Our previous study also found HBsAg‐positive DLBCL patients had worse 1‐ and 3‐year OS/PFS.^27^ All of the above studies suggested that HBsAg positive was a poor prognostic factor for HBV‐related DLBCL. However, in this study, we did not find significant differences between HBV‐positive DLBCL and HBV‐negative DLBCL in terms of age, sex, stage, IPI score, and OS, which contradicted the results of the above studies. We considered this to be related to several factors: (1) Thirteen patients (25%) from the HBV‐positive group of this study participated in a clinical study initiated by our academic center on November 30, 2020 (unpublished data). This clinical study was aimed at evaluating the efficacy and safety of chidamide for maintenance therapy in HBV‐positive DLBCL patients. Of the 37 HBV‐positive DLBCL patients collected so far, 22 were treated with chidamide, of which 13 were included in this study. The study is still ongoing and we will follow up with further validation of the role of chidamide in HBV‐positive DLBCL. (2) This study collected patient data from the last 5 years, and compared to the previous period, there have been better developments in treatment regimens and radiotherapy techniques, and more widespread use of rituximab, all of which could further improve the survival benefit for patients with lymphoma. Moreover, our study also showed that CLL/SLL patients had an even slightly higher rate of HBV infection than DLBCL. However, there were no significant differences in age, stage, and PFS between HBV‐positive and HBV‐negative CLL/SLL. Overall, no studies have been conducted nationally or internationally to further compare the clinical characteristics and survival differences among patients with different HBV infection statuses in aggressive and indolent B‐NHL. Although no statistical difference was observed in our findings, a trend of relatively higher HBV infection rates in DLBCL and CLL/SLL has been observed, which can be explored by further relevant clinical and basic studies in the future, and there may be new findings.

Our research has some limitations. First of all, it is a single center, retrospective analysis that inevitably had a certain selection bias. Second, this study focused on HBV and did not consider other confounding factors, such as whether patients had other diseases with immune abnormalities, because drug treatment of such diseases could also cause hepatitis B virus reactivation. Third, this study compared different NHL subtypes separately, and the small sample sizes for some subtypes may have had an impact on the results. Fourth, regrettably, there was insufficient information on HBV‐DNA quantification in this study to demonstrate the correlation between occult and apparent HBV infections and B‐NHL subtypes.

## 5 CONCLUSION

In summary, our research suggests that there is a correlation between HBV infection and NHL, that the higher prevalence of HBV infection in B‐NHL should not be ignored, and that further studies are necessary in the future to explore the association between HBV infection and different pathological subtypes of B‐NHL. It may be possible to discover the unique clinicopathological, prognostic, and genetic features of HBV‐positive lymphoma patients, further reveal the key functional molecules and core mechanisms associated with HBV and lymphoma, and provide new target information or drug repositioning strategies for clinical treatment, thus providing an important basis for the selection of therapeutic strategies for HBV‐positive lymphoma patients.

## AUTHOR CONTRIBUTIONS

**Zhaoxia Li:** Conceptualization (equal); formal analysis (equal); writing – original draft (equal). **Wei Guo:** Data curation (equal). **Yangzhi Zhao:** Data curation (equal). **Haotian Wang:** Investigation (equal). **Jing Guo:** Investigation (equal). **Zhe Li:** Investigation (equal). **Bowen Wang:** Investigation (equal). **Luming Cao:** Investigation (equal). **Jihong Xu:** Investigation (equal). **Ken H. Young:** Conceptualization (equal); writing – review and editing (equal). **Ou Bai:** Conceptualization (equal); writing – review and editing (equal).

## FUNDING INFORMATION

This work was supported by Department of Science and Technology of Jilin Province (20220402064GH).

## CONFLICT OF INTEREST STATEMENT

All authors declare no conflict of interest.

## ETHICS STATEMENT

This retrospective study protocol was conducted in conformity with the Declaration of Helsinki (as revised in Edinburgh 2000) and was approved by the ethics committee of the first hospital of Jilin University (approval number: 2024–482). As this study is a retrospective study and patient anonymization, the Ethics Committee has determined to be exempt from signing informed consent forms.

## Data Availability

The datasets created and examined during the current work are not publicly accessible due to privacy and ethical considerations, but they are available from the corresponding author upon justifiable request.
